# Supplementary material for: Functional expression of the nitrogenase Fe protein in transgenic rice
Source: Commun Biol. 2022 Oct 5;5:1006. doi: 10.1038/s42003-022-03921-9 (PMC9534833; doi:10.1038/s42003-022-03921-9)
Supplement: Supplementary file 2 — Supplementary Information [file 42003_2022_3921_MOESM2_ESM.pdf]

SUPPLEMENTARY INFORMATION

Title

Functional expression of the nitrogenase Fe protein in transgenic rice

Authors

Can Baysal[1,2≠], Stefan Burén[3,4≠], Wenshu He[1], Xi Jiang[3,4], Teresa Capell[1], Luis M. Rubio[3,4]* and Paul Christou[1,5]*

Affiliations

[1]Department of Plant Production and Forestry Science, University of Lleida-Agrotecnio CERCA Center, Av. Alcalde Rovira Roure, 191, 25198 Lleida, Spain

[2]Current address: Department of Genetics, Cell Biology and Development, University of Minnesota, St. Paul, MN, USA

[3]Centro de Biotecnología y Genómica de Plantas, Universidad Politécnica de Madrid (UPM) - Instituto Nacional de Investigación y Tecnología Agraria y Alimentaria (INIA), Campus Montegancedo UPM, 28223 Pozuelo de Alarcón (Madrid), Spain

[4]Departamento de Biotecnología-Biología Vegetal, Escuela Técnica Superior de Ingeniería Agronómica, Alimentaria y de Biosistemas, Universidad Politécnica de Madrid, 28040 Madrid, Spain

[5]ICREA, Catalan Institute for Research and Advanced Studies, Passeig Lluís Companys 23, 08010 Barcelona, Spain

Corresponding Authors

*Paul Christou

Email: paul.christou@udl.cat

*Luis M. Rubio

Email: lm.rubio@upm.es

≠ These authors contributed equally to this work

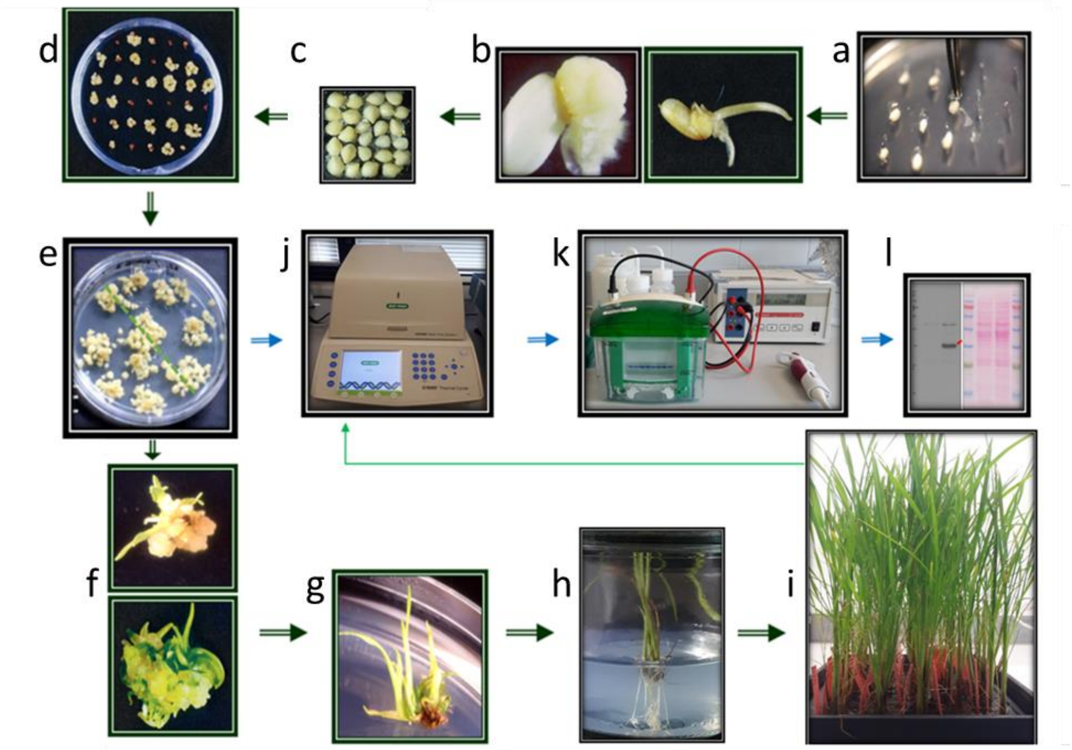

Supplementary Fig. 1 Rice transformation procedure, recovery and analysis of transgenic callus and plants. **(a)** Seed germination. **(b)** Six-day-old mature zygotic embryos. **(c)** Isolated embryos for gene delivery. **(d)** Embryos after bombardment growing under hygromycin selection. **(e)** Embryogenic callus after two rounds (15 days) of hygromycin selection. **(f, g)** Regenerating callus. **(h)** Root formation. **(i)** Transgenic plants (2-month-old T0 clonal sibling plants of line Ht200). **(j–l)** Expression analysis at the mRNA and protein levels of callus and corresponding regenerated plants.

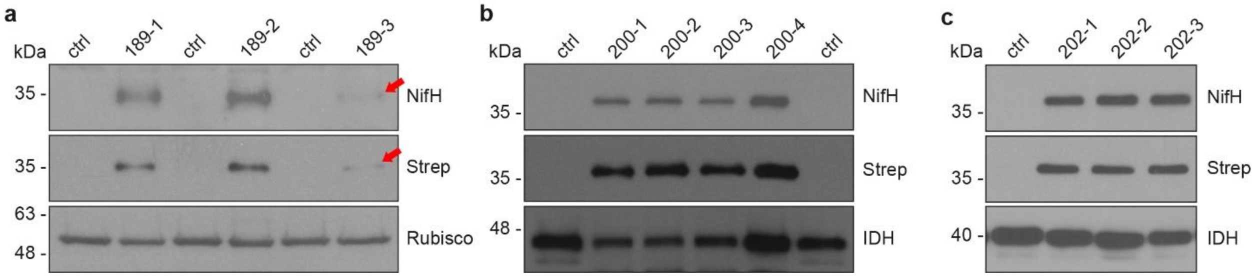

Supplementary Fig. 2 *Os*NifH^{Ht} accumulation in rice plants. Immunoblot analysis of *Os*NifH^{Ht} showing accumulation in individual plants regenerated from callus lines **(a)** Ht189, **(b)** Ht200 and **(c)** Ht202 using antibodies against NifH and the Strep tag (Strep). Red arrows show *Os*NifH^{Ht} weakly expressed in plant 3 regenerated from line Ht189. Antibodies against RuBisCO or IDH were used as loading controls. Ctrl lanes shows non-transformed callus and plant lines. Uncropped immunoblots are shown in **Supplementary Fig. 13-15**.

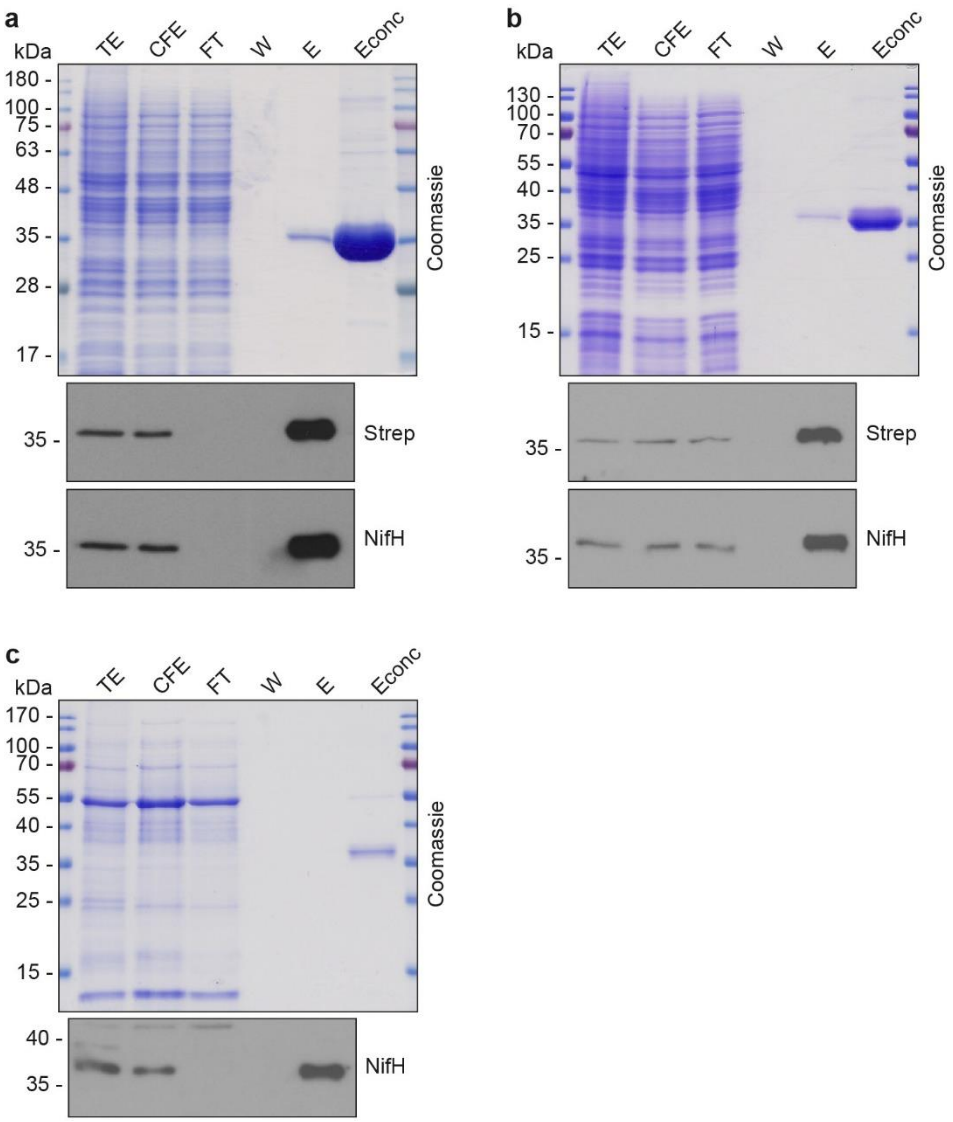

Supplementary Fig. 3 STAC purification of *Os*NifH^{Ht} protein targeted to rice mitochondria.

Purification of *Os*NifH^{Ht} from **(a)** Ht200 callus, **(b)** Ht202 callus and **(c)** Ht200 plants. Fractions were analyzed by SDS-PAGE followed by Coomassie staining or immunoblotting. TE = total extract, CFE = soluble cell-free extract, FT = flow-through fraction, W = wash fraction, E = elution fraction. Econc = concentrated elution fraction (final sample collected). Uncropped immunoblots for the purification processes are shown in **Supplementary Fig. 16**.

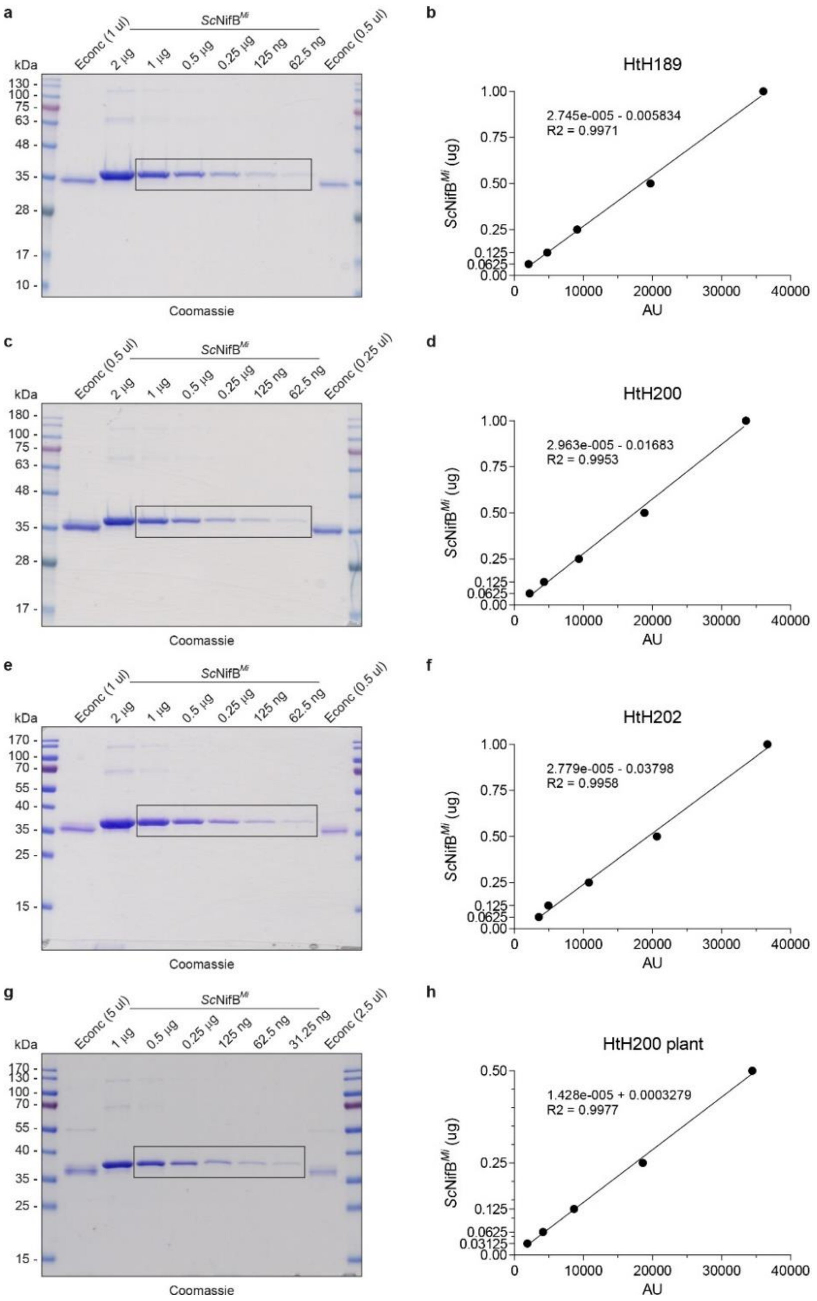

Supplementary Fig. 4 Quantification of *Os*NifH^{Ht} isolated from rice callus and plants. SDS-PAGE and Coomassie staining of the final *Os*NifH^{Ht} protein samples (Econc) isolated from **(a)** Ht189 callus, **(c)** Ht200 callus, **(e)** Ht202 callus and **(g)** Ht200 plants together with the indicated amounts of purified *Sc*NifB^{Mi} protein (quantified using the BCA protein assay)[1]. The *Sc*NifB^{Mi} samples enclosed by black squares were used to create a standard curve for quantification of the *Os*NifH^{Ht} proteins isolated from **(b)** Ht189 callus, **(d)** Ht200 callus, **(f)** Ht202 callus and **(h)** Ht200 plants. The intercept and slope for the *Sc*NifB^{Mi} standard curve and the R^2 fit to the curve are indicated.

MRQIAIYGKG GIGK**STTTQN TVAALAEAGR KCFIVGCDPK ADSTR**LILHV
K**AQSTVMHLA AERGAVEDLD LDEVMLVGFG GIKCVESGGP EPGVGCAGR**G
VITAINFLEE NGAFDDDLDY VFYDVLGDVV CGGFAMPIR**E GKAQEIYIVT**
SGEMMAMYAA NNISKGILKY AHSGGVRLGG LICNSR**NVDN ERELIEALAE**
KLGTQMIHFL PRNNIVQEAE LR**RMTVIEYA PDHPMADEYR** TLAKKIEENR
KLSIPTPLTM DELEQLLVEY GIMKPEEVA

Supplementary Fig. 5 Mass spectrometry analysis of purified *Os*NifH^{Ht} protein. Bold red letters indicate the peptides from *Os*NifH^{Ht} (isolated from Ht189 callus) that were identified by mass spectrometry.

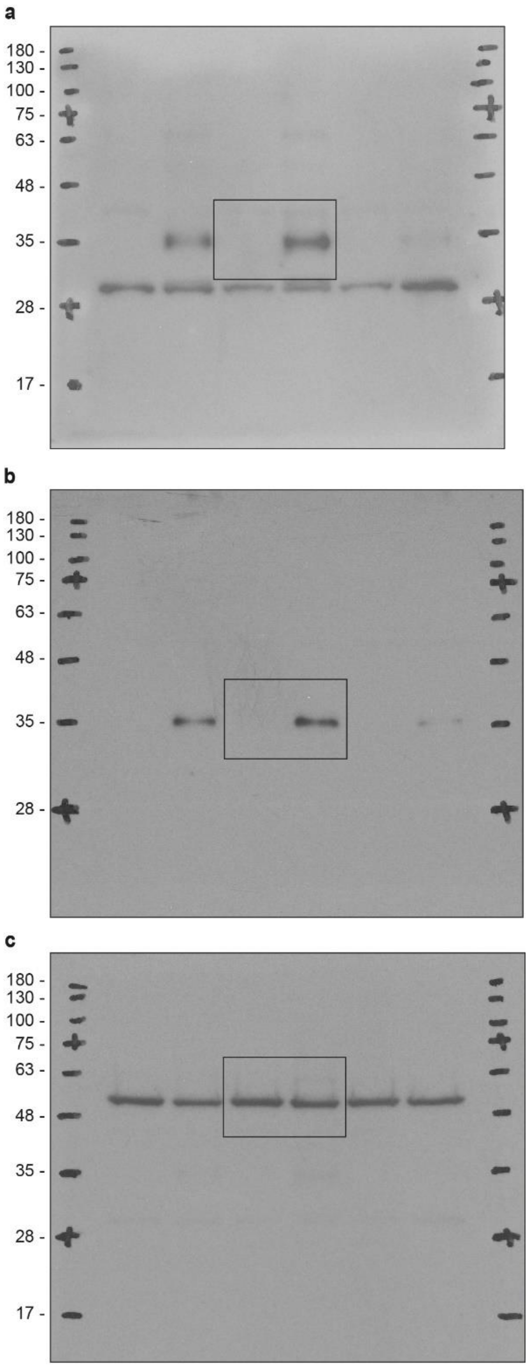

Supplementary Fig. 6 Uncropped immunoblots (Ht189 plant) shown in Fig. 1c. (**a**) NifH. (**b**) Strep. (**c**) RuBisCO.

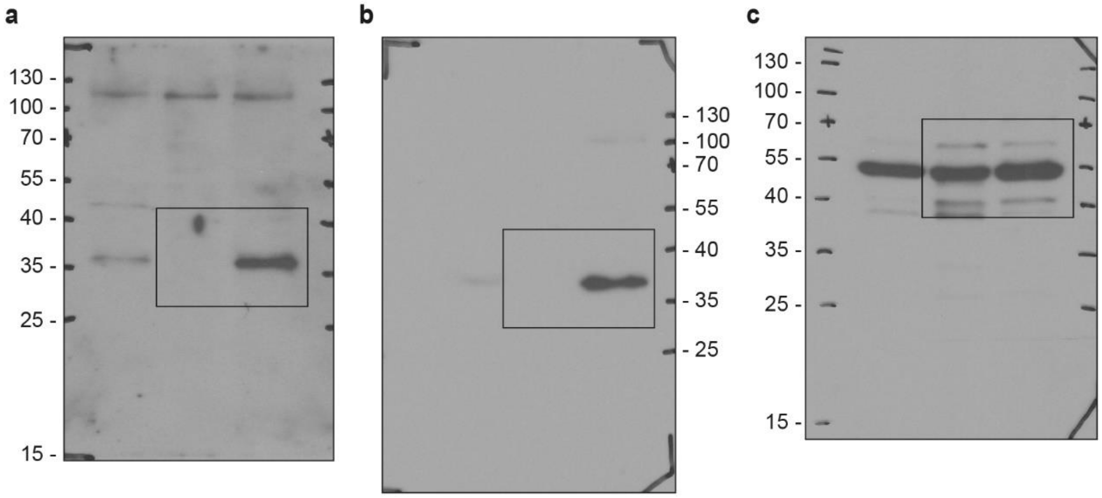

Supplementary Fig. 7 Uncropped immunoblots (Ht200 plant) shown in Fig. 1c. (**a**) NifH. (**b**) Strep. (**c**) RuBisCO.

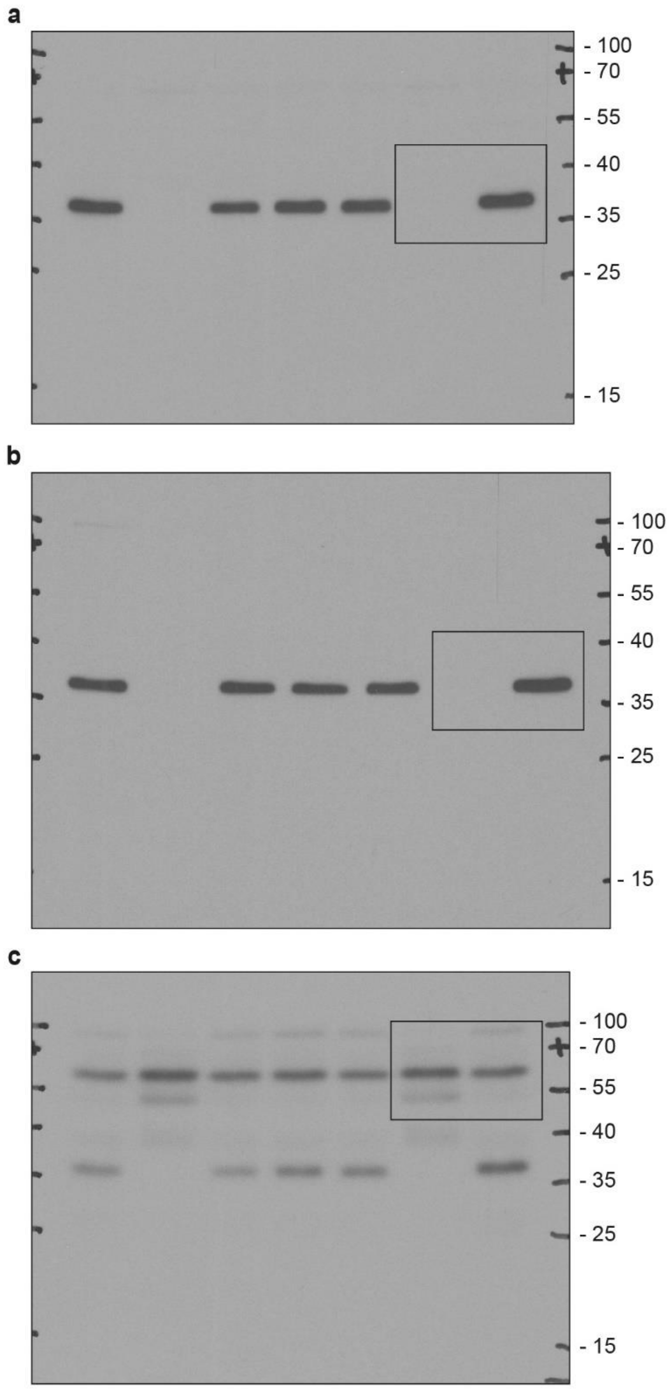

Supplementary Fig. 8 Uncropped immunoblots (Ht202 plant) shown in Fig. 1c. (**a**) NifH. (**b**) Strep. (**c**) RuBisCO.

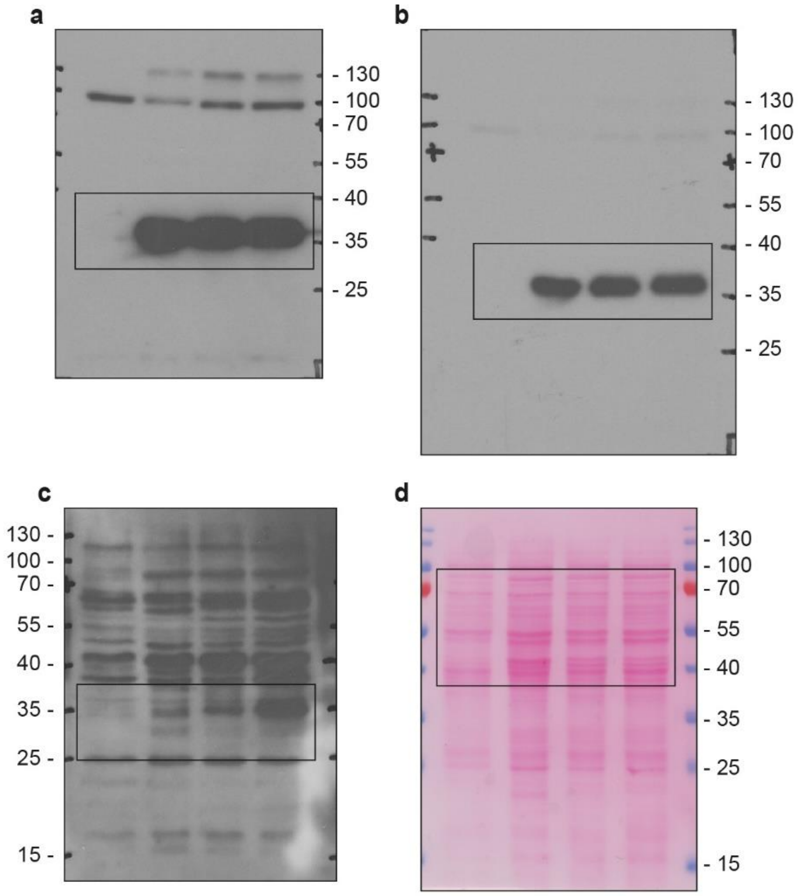

Supplementary Fig. 9 Uncropped immunoblots shown in Fig. 1d. (**a**) NifH. (**b**) Strep. (**c**) NifM. (**d**) Ponceau.

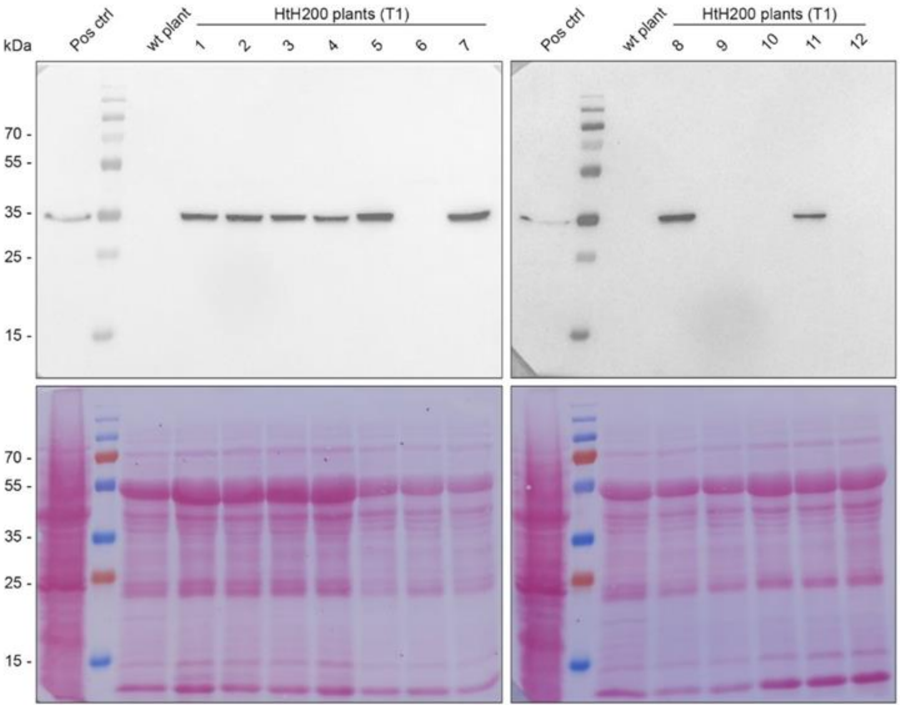

Supplementary Fig. 10 Uncropped immunoblots shown in Fig. 1e.

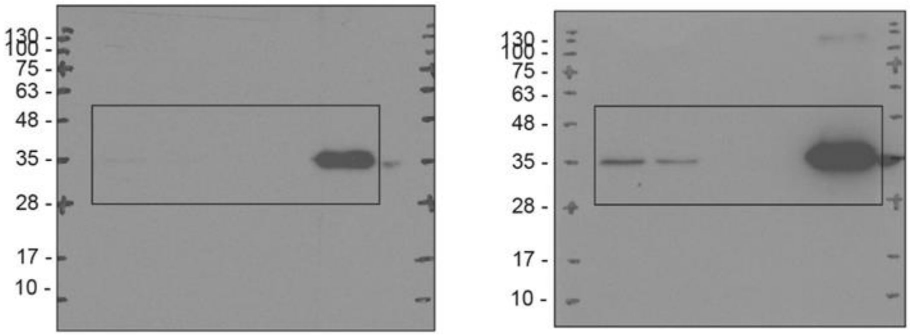

Supplementary Fig. 11 Uncropped immunoblots shown in Fig. 2a.

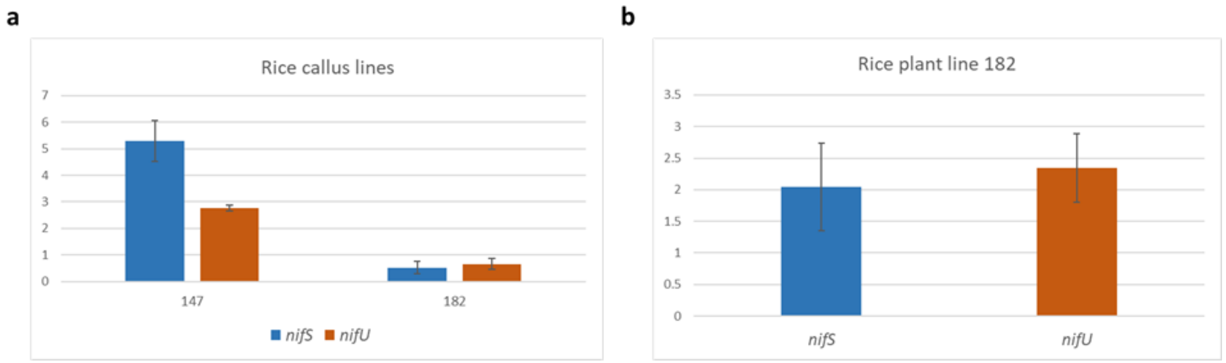

Supplementary Fig. 12 Relative mRNA expression (qRT-PCR) of *Azotobacter vinelandii nifS* and *nifU* in two independent rice callus lines (a) and regenerated plant line 182 (b). Callus line 147 failed to regenerate**.** Data (normalized to *OsActin* mRNA) are means ± SD (n = 3 technical replicates). The sequences of the *nifS* and *nifU* genetic components are listed in Supplementary Table 1. Primers used for *nifS and nifU* vector construction and quantitative real-time PCR are listed in Supplementary Table 2.

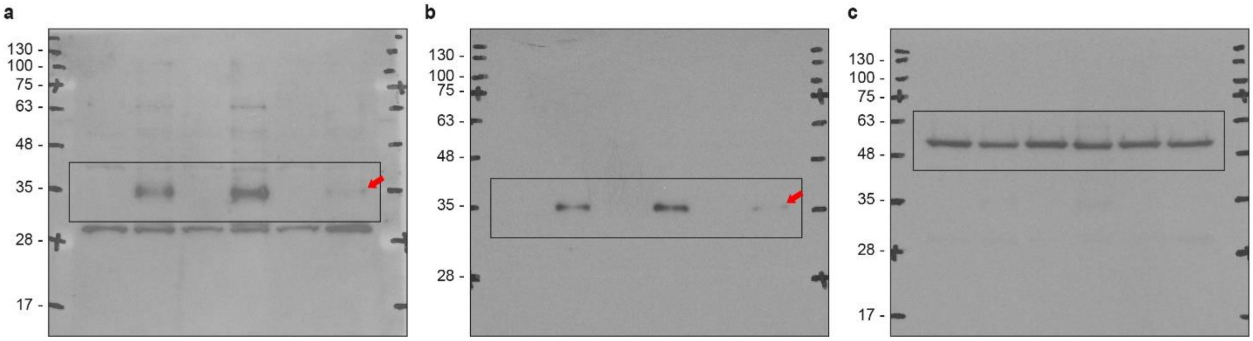

Supplementary Fig. 13 Uncropped immunoblots shown in Supplementary Fig. 2a. (**a**) NifH. (**b**) Strep. (**c**) RuBisCO.

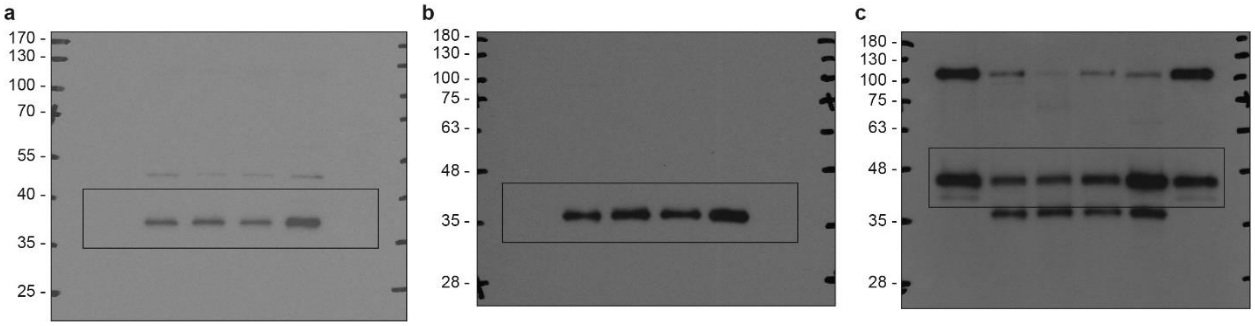

Supplementary Fig. 14 Uncropped immunoblots shown in Supplementary Fig. 2b. (**a**) NifH. (**b**) Strep. (**c**) IDH.

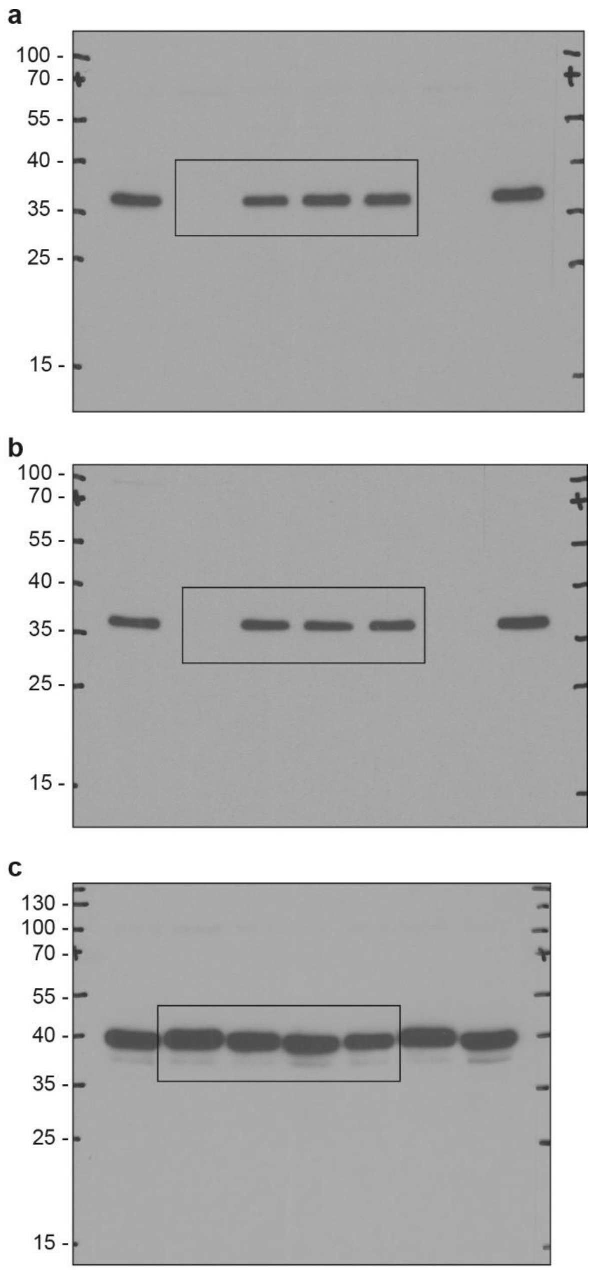

Supplementary Fig. 15 Uncropped immunoblots shown in Supplementary Fig. 2c. (**a**) NifH. (**b**) Strep. (**c**) IDH.

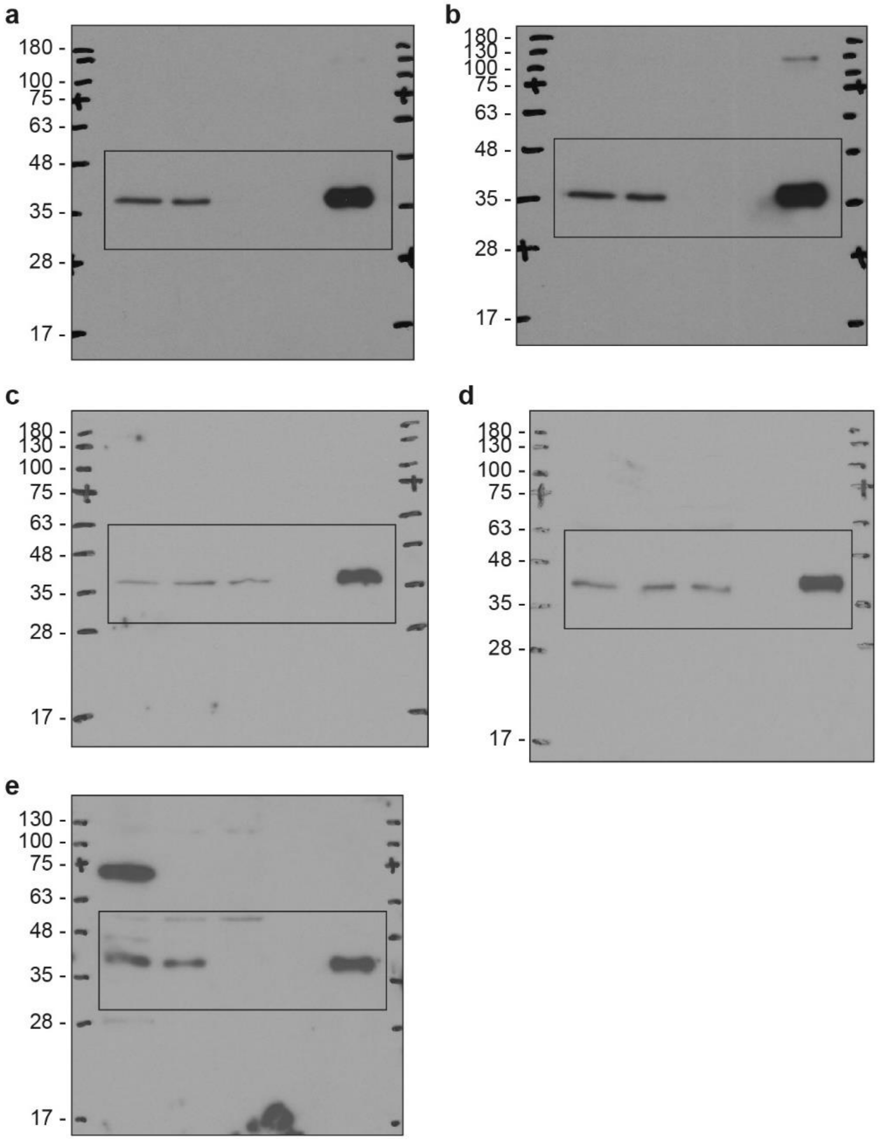

Supplementary Fig. 16 Uncropped immunoblots shown in Supplementary Fig. 3. (**a**) Supplementary Fig. 3a (Strep). (**b**) Supplementary Fig. 3a (NifH). (**c**) Supplementary Fig. 3b (Strep). (**d**) Supplementary Fig. 3b (NifH). (**e**) Supplementary Fig. 3c (NifH).

Supplementary Tables

Supplementary Table 1 Plant expression vectors used and sequences of genetic constructs.

**Plant expression vectors used**			
**Plasmid backbone**	**Expressed proteins**	**Promoter / Terminator**	**Size (kDa)**<br>**FL.** **P.**
pUC57	Cox4-TS-NifH^{Ht}	p*ZmUbi1*+1st intron / tNos	37.0 33.5
pUC57	SU9-NifM^{Av}	p*OsActin* / tNos	40.4 33.0
pUC57	SU9-NifS^{Av}	p*ZmUbi1*+1st intron/tADH1	51.3 44.0
pUC57	SU9-Nifu^{Av}	p*ZmUbi1*+1st intron/tCYC1	40.8 33.6

**Sequences of genetic constructs**
**Cox4:** MLSLRQSIRFFKPATRTLCSSRYLLQQKP
**Su9:** MASTRVLASRLASQMAASAKVARPAVRVAQVSKRTIQTGSPLQTLKRTQMTSIVNATTRQAFQKRAYSS
**NifH^{Ht}:**<br>MRQIAIYGKGGIGKSTTTQNTVAALAEAGRKCFIVGCDPKADSTRLILHVKAQSTVMHLAAERGAVEDLDLDEVMLVGFGGIKCV<br>ESGGPEPGVGCAGRGVITAINFLEENGAFDDDLDYVFYDVLGDVVCGGFAMPIREGKAQEIYIVTSGEMMAMYAANNISKGILKY<br>AHSGGVRLGGLICNSRNVDNERELIEALAEKLGTQMIHFLPRNNIVQEAELRRMTVIEYAPDHPMADEYRTLAKKIEENRKLSIPTP<br>LTMDELEQLLVEYGIMKPEEVA*
**NifM^{Av}:**<br>MASERLADGDSRYYLLKVAHEQFGCAPGELSEEQLQQADRIIGRQRHIEDAVLRSPDAIGVVIPPSQLEEAWAHIASRYESPEALQQ<br>ALDAQALDAAGMRAMLARELRVEAVLDCVCAGLPEISDTDVSLYYFNHAEQFKVPAQHKARHILVTINEDFPENTREAARTRIETI<br>LKRLRGKPERFAEQAMKHSECPTAMQGGLLGEVVPGTLYPELDACLFQMARGELSPVLESPIGFHVLYCESVSPARQLTLEEILPRL<br>RDRLQLRQRKAYQRKWLESLLQQNATLENLAHG*
**NifS^{Av}:**<br>MRPADVYLDNNATTRVDDEIVQAMLPFFTEQFGNPSSLHSFGNQVGMALKKARQSVQKLLGAEHDSEIVFTSCGTESDSTAILSAL<br>KAQPERKTVITTVVEHPAVLSLCDYLASEGYTVHKLPVDKKGRLDLEHYASLLTDDVAVVSVMWANNETGTLFPIEEMARLADD<br>AGIMFHTDAVQAVGKVPIDLKNSSIHMLSLSGHKLHAPKGVGVLYLRRGTRFRPLLRGGHQERGRRAGTENAASIIGLGVAAERAL<br>QFMEHENTEVKRLRDKLEAGILAVVPHAFVTGDPDNRLPNTANIAFEYIEGEAILLLLNKVGIAASSGSACTSGSLEPSHVMRAMDI<br>PYTAAHGTVRFSLSRYTTEEEIDRVIREVPPIVAQLRKLSPYWSGNGPVEDPGKAFAPVYG*
**NifU^{Av}:**<br>MRPWDYSEKVKEHFYNPKNAGAVEGANAIGDVGSLSCGDALRLTLKVDPETDVILDAGFQTFGCGSAIASSSALTEMVKGLTLDE<br>ALKISNQDIADYLDGLPPEKMHCSVMGREALQAAVANYRGETIEDDHEEGALICKCFAVDEVMVRDTIRANKLSTVEDVTNYTKA<br>GGGCSACHEAIERVLTEELAARGEVFVAAPIKAKKKVKVLAPEPAPAPVAEAPAAAPKLSNLQRIRRIETVLAAIRPTLQRDKGDVEL<br>IDVDGKNVYVKLTGACTGCQMASMTLGGIQQRLIEELGEFVKVIPVSAAAHAQMEV*
**FL**, full-length; **P,** processed by removal of mitochondria targeting peptide. **TS,** tween-strep.<br>**Cox4,** *Saccharomyces cerevisiae c*ytochrome c oxidase subunit 4 mitochondrial targeting peptide.<br>**Su9,** *Neurospora crassa* subunit 9 mitochondrial targeting peptide.

Supplementary Table 2 Primers used for vector construction and quantitative real-time PCR analysis.

Gene	Primers used for quantitative Real-Time PCR analysis
NifH^{Ht} – (F)	5'-ACGCTCATTCTGGTGGTGTT-3'
NifH^{Ht} – (R)	5'-CCATTGGATGATCTGGGGCA-3'
NifM^{Av} – (F)	5'-TGCAAGGTGGGTTTGTTAGGTG-3'
NifM^{Av} – (R)	5'-GTCTTGCTGGACTGACGGAT-3'
NifS^{Av} – (F)	5'-GCAGACAAAATGGCGGTTGA-3'
NifS^{Av} – (R)	5'-ACCCTTCCAGTTTGCATTCCT-3'
NifU^{Av} – (F)	5'-CGAAACAGTCTTGGCTGCAA-3'
NifU^{Av} – (R)	5'-CAGCGGAGACTGGGATAAC-3'
OsActin – (F)	5'-TCATGTCCCTCACAATTTCC-3'
OsActin – (R)	5'-GACTCTGGTGATGGTGTCAGC-3'
	**Primers used for vector construction**
pOsActin – (F) (*nifM*)	5'-TAAGCAGGTACCTAGCTAGCATACTCGAGGT -3'
pOsActin – (R) (*nifM*)	5'-TGCTTATCTAGACTTCTACCTACAAAAAAGC-3'
NifM^{Av} – (F)	5'-TAAGCATCTAGAATGGCCTCCACTCGTGTCC-3'
NifM^{Av} – (R)	5'-TGCTTAGTCGACGATCTAGTAACATAGATGA-3'
pZmUbi1 – (F) (*nifH*)	5'-TAAGCAGGATCCGGAGTGCAGTGCAGCGTGA-3'
pZmUbi1 – (R) (*nifH*)	5'-TGCTTACTGCAGAAGTAACACCAAACAACAG-3'
NifH^{Ht} – (F)	5'-TAAGCACTGCAGAATGCTTTCACTTAGACAA-3'
NifH^{Ht} – (R)	5'-TGCTTAGCATGCACATACAAATGGACGAACG-3'
pZmUbi1– (F) (*nifS*)	5'-TAAGCAGAATTCTGCAGTGCAGCGTGACCCG-3'
pZmUbi1– (R) (*nifS*)	5'-TGCTTAGGATCCCTGCAGAAGTAACACCAAA-3'
NifS^{Av} – (F)	5'-TAAGCAGGATCCATGGCCTCCACTCGTGTCC-3'
NifS^{Av} – (R)	5'-TGCTTAAAGCTTGAGCGACCTCATGCTATAC-3'
pZmUbi1– (F) (*nifU*)	5'-TAAGCAGAATTCTGCAGTGCAGCGTGACCCG-3'
pZmUbi1 – (R) (*nifU*)	5'-TGCTTAGAGCTCCTGCAGAAGTAACACCAAA-3'
NifU^{Av} – (F)	5'-TAAGCAGAGCTCATGGCCTCCACTCGTGTCC-3'
NifU^{Av} – (R)	5'-TGCTTAGGATCCCTTCGAGCGTCCCAAACC-3'

Supplementary References

1. Burén, S. et al. Biosynthesis of the nitrogenase active-site cofactor precursor NifB-co in Saccharomyces cerevisiae. *Proceedings of the National Academy of Sciences of the United States of America*, **116**, 25078–25086 (2019).
